# Late Pleistocene fishes of the Tennessee River Basin: an analysis of a late Pleistocene freshwater fish fauna from Bell Cave (site ACb-2) in Colbert County, Alabama, USA

**DOI:** 10.7717/peerj.1648

**Published:** 2016-02-02

**Authors:** Stephen J. Jacquemin, Jun A. Ebersole, William C. Dickinson, Charles N. Ciampaglio

**Affiliations:** 1 Lake Campus, Wright State University, Celina, OH, United States; 2 Collections Department, McWane Science Center, Birmingham, AL, United States; 3 412 Nottingham Court, Maryville, TN, United States

**Keywords:** Long term assemblage change, North America, Freshwater fish biogeography

## Abstract

The Tennessee River Basin is considered one of the most important regions for freshwater biodiversity anywhere on the globe. The Tennessee River Basin currently includes populations of at least half of the described contemporary diversity of extant North American freshwater fishes, crayfish, mussel, and gastropod species. However, comparatively little is known about the biodiversity of this basin from the Pleistocene Epoch, particularly the late Pleistocene (∼10,000 to 30,000 years B.P.) leading to modern Holocene fish diversity patterns. The objective of this study was to describe the fish assemblages of the Tennessee River Basin from the late Pleistocene using a series of faunas from locales throughout the basin documented from published literature, unpublished reports, and an undocumented fauna from Bell Cave (site ACb-2, Colbert County, AL). Herein we discuss 41 unequivocal taxa from 10 late Pleistocene localities within the basin and include a systematic discussion of 11 families, 19 genera, and 24 identifiable species (28 unequivocal taxa) specific to the Bell Cave locality. Among the described fauna are several extirpated (e.g., Northern Pike *Esox lucius*, Northern Madtom *Noturus stigmosus*) and a single extinct (Harelip Sucker *Moxostoma lacerum*) taxa that suggest a combination of late Pleistocene displacement events coupled with more recent changes in habitat that have resulted in modern basin diversity patterns. The Bell Cave locality represents one of the most intact Pleistocene freshwater fish deposits anywhere in North America. Significant preservational, taphonomic, sampling, and identification biases preclude the identification of additional taxa. Overall, this study provides a detailed look into paleo-river ecology, as well as freshwater fish diversity and distribution leading up to the contemporary biodiversity patterns of the Tennessee River Basin and Mississippi River Basin as a whole.

## Introduction

**Figure 1:**
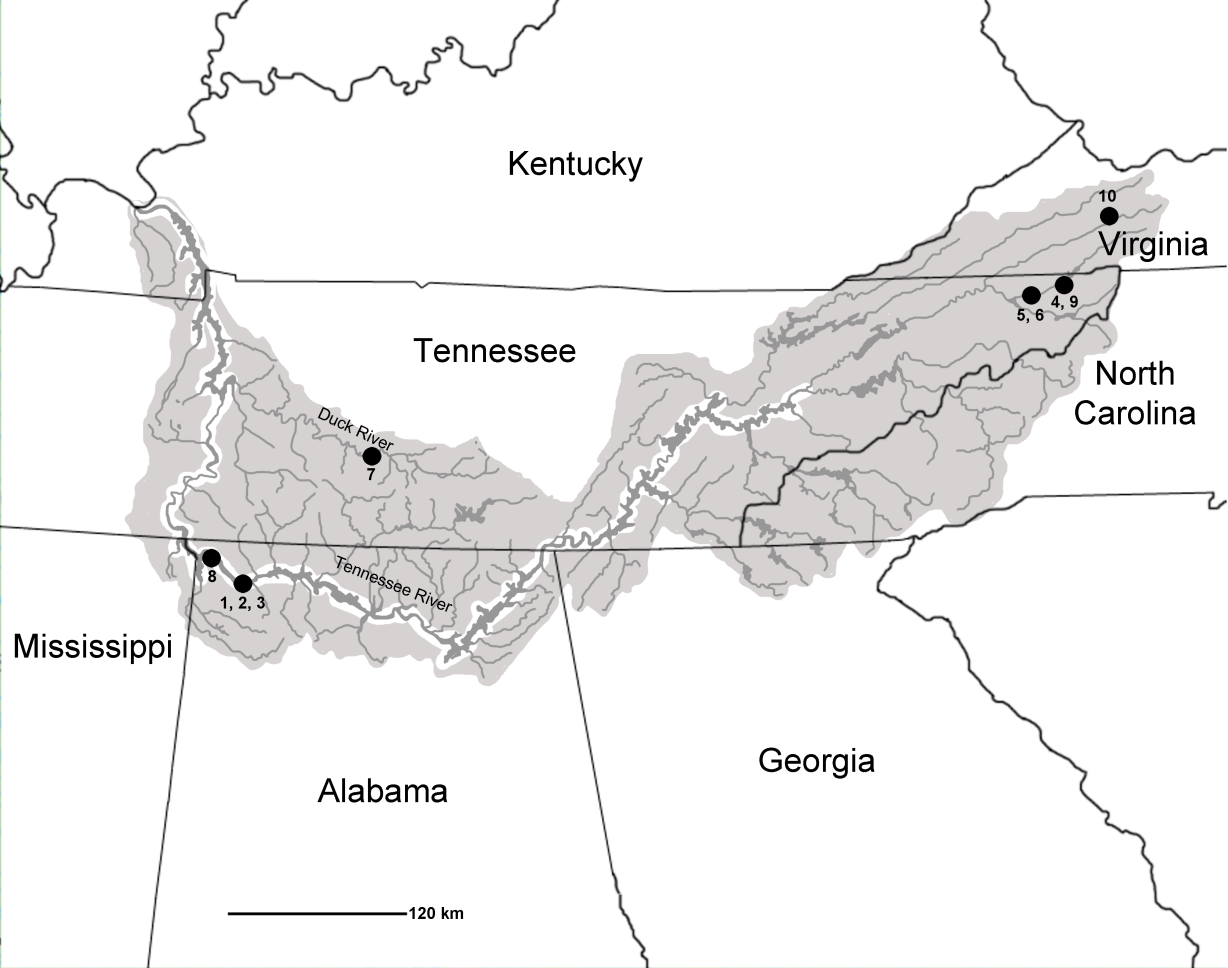
Map of Tennessee River Basin. Shaded areas show the extent of the Basin. Listed late Pleistocene localities are as follows: 1, Bell Cave (site ACb-2), Colbert County, AL.; 2, site ACb-3, Colbert County, AL; 3, site ACb-5, Colbert County, AL; 4, Appalachian Caverns, Sullivan County, TN; 5, Baker Bluff Cave, Sullivan County, TN; 6, Beartown Cave, Sullivan County, TN; 7, Cheek Bend Cave, Maury County, TN; 8, Dust Cave, Lauderdale County, AL; 9, Guy Wilson Cave, Sullivan County, TN; 10, Saltville, Smythe County, VA.

Over the past 70 years, several comprehensive synopses have been published on North American Quaternary freshwater fishes (see Miller, 1959; Miller, 1965; Uyeno & Miller, 1963), with the last major works written by Smith (1981) and Cavender (1986). Absent from these reviews, however, are reports of the Tennessee River Basin as a whole or any reports of late Pleistocene fish remains from many southeastern localities (in particular, sites within Alabama and Tennessee). This has important implications given that the southeastern United States is one of the largest hotspots of freshwater biodiversity, with the modern Tennessee River Basin being home to approximately 240 fish species (Appendix S2; National Water Quality Assessment Program, 2015; Etnier & Starnes, 1993; Boschung & Mayden, 2004; Page & Burr, 2011). The Tennessee River Basin encompasses an aerial extent that covers more than 105,000 km^2^ and stretches across seven states: Alabama, Georgia, Kentucky, Mississippi, North Carolina, Tennessee, and Virginia (Etnier & Starnes, 1993; Fig. 1). The diversity of fishes and habitats in this geographic area warrants additional study of the fossil record to provide a better understanding of the region’s paleoecology, biogeography, and evolutionary history. The objective of this study was to concatenate and add to prior research to provide a more comprehensive understanding of the Pleistocene ichthyofaunal record of the Tennessee River Basin.

Known Pleistocene sites with fish occurrences from the Tennessee River Basin include sites ACb-2 (reported herein), ACb-3 (4 unequivocal taxa; identified by authors), ACb-5 (1 taxon; identified by authors); and Little Bear Cave (unidentified fish taxa; Womochel & Barnett, 1980) in Colbert County, AL; Dust Cave (7 unequivocal fish taxa; Walker, 1998) in Lauderdale County, AL; Appalachian Caverns (5 unequivocal taxa; identified by authors), Baker Bluff Cave (14 unequivocal fish taxa; Guilday et al., 1978), Beartown Cave (1 taxon; identified by authors), and Guy Wilson Cave (3 unequivocal taxa; identified by authors) in Sullivan County, TN; Cheek Bend Cave (25 unequivocal fish taxa; Dickinson, 1986) in Maury County, TN; and Saltville (1 taxon; McDonald & Bartlett, 1983; 3 additional unequivocal taxa; Dickinson, 1986) in Smythe County,VA (Fig. 1). While these sites do represent numerous locales across the Tennessee River Basin, none of the described sites include the mainstem of the Tennessee River and only two sites (Baker Bluff Cave and Cheek Bend Cave) had reported more than a few taxa. The relatively low number of taxa and lack of mainstem habitat signifies a need for additional samples to better assess the diversity of the region during this time period.

During the summers of 1984 and 1987, personnel from the former Red Mountain Museum (Birmingham, AL) and the Alabama Museum of Natural History (Tuscaloosa) conducted extensive excavations within Bell Cave (site ACb-2; Colbert County, Alabama), located along the south bank of the lower reaches of the mainstem Tennessee River. The cave’s locale along the mainstem Tennessee River and position within the basin make it potentially one of the most significant North American locations for documenting Pleistocene freshwater fish biodiversity. To date, excavations of Bell Cave have resulted in the recovery of nearly 4,000 cataloged lots of late Pleistocene faunal remains from four distinct stratigraphic levels within the cave. However, to this point, no formal description of the Bell Cave ichthyofauna has been undertaken.

Formal descriptions of other faunas captured from Bell Cave have included amphibians and reptiles (Holman, Bell & Lamb, 1990; Holman, 1995; Jasinski, 2013), birds (Parmalee, 1992), and mammals ranging in size from rodents to extinct megafauna (Bell, 1985; Churcher et al., 1989; Martin & Prince, 1990; Parmalee & Graham, 2002; McDonald, 2003; Ruez, 2008; Ebersole & Ebersole, 2011). Specific to the fishes, however, only brief mention have appeared in the literature and only as ancillary information. Churcher et al. (1989: 1214), for example, mentioned at least “20 fish” taxa (based on preliminary identifications by WC Dickinson) among the Bell Cave faunas while Parmalee & Graham (2002) simply reported the presence of “fish” within the cave. Parmalee (1992) was the first to mention specific fish taxa from Bell Cave, noting the presence of Sturgeon *Acipenser*, Drum *Aplodinotus*, Catfish *Ictalurus*, and Redhorse *Moxostoma*, but did not cite any cataloged specimens or provide any systematic material or comparisons. Formal descriptions of the fishes from Bell Cave will hopefully help fill a gap in our understanding of regional diversity during this time period.

The purpose of this study is to expand our understanding of late Pleistocene fish diversity in the Tennessee River basin by closely examining existing faunal lists as well as describing the 235 cataloged lots of Bell Cave fish material to develop our understanding of the fish assemblages during this time period. These fish remains represent one of the largest Pleistocene fish faunas reported from North America, the largest from the Tennessee River Basin, and the first published from the state of Alabama. The overarching comparison of the Bell Cave fauna to others within the Tennessee River Basin not only serves to fill a regional gap in terms of knowledge of late Pleistocene fish occurrences, but also provides valuable insights into the biogeography and paleoecology of these fish taxa within the basin during the late Pleistocene.

## Materials & Methods

Late Pleistocene faunal lists from localities located within the boundaries of the Tennessee River Basin were recorded based on both published and unpublished accounts. All specimens reported from unpublished localities were directly examined by one or more of the authors. While the primary focus of this manuscript is on the formal description of the Bell Cave fish fauna, all known (nine additional) late Pleistocene localities are reviewed in this study as a means to compare with the Bell Cave fauna and to provide a larger view of the late Pleistocene fishes within the Tennessee Basin. These late Pleistocene localities were chosen based on the following criteria: (1) locality had a described late Pleistocene fish fauna (published or unpublished); (2) the locality was within the recognized boundary of the Tennessee River Basin; and (3) the site had described faunal remains that were late Wisconsinan in age or fall within the age range of 10,000–30,000 years before present. The fish faunas reviewed herein are the result of a search for sites meeting these criteria and derive localities that span the eastern to western extents of Tennessee River Basin, thus providing a representative overview of the late Pleistocene fishes known from the entirety of the Basin. Localities that fit these criteria are as follows, with general locations presented in Fig. 1:

1. Site ACb-2 Bell Cave, Colbert County, AL—Source: This study.
2. Site ACb-3, Colbert County, AL—Source: This study.
3. Site ACb-5, Colbert County, AL—Source: This study.
4. Appalachian Caverns, Sullivan County, TN—Source: This study.
5. Baker Bluff Cave, Sullivan County, TN—Source: Guilday et al., 1978.
6. Beartown Cave, Sullivan County, TN—Source: Guilday, Hamilton & Parmalee, 1975.
7. Cheek Bend Cave, Maury County, TN—Source: Dickinson, 1986.
8. Dust Cave, Lauderdale County, AL—Source: Walker, 1998.
9. Guy Wilson Cave, Sullivan County, TN—Source: This study.
10. Saltville, Smythe County, VA—Source: McDonald & Bartlett, 1983; Dickinson, 1986.

The examined material for this study was recovered from Bell Cave in Colbert County, AL during the summers of 1984 and 1987 by personnel from the former Red Mountain Museum in Birmingham, AL and the Alabama Museum of Natural History in Tuscaloosa. Beginning in 1984, a series of five 1.0 m^2^ units were excavated within the main room of Bell Cave with matrix removed one stratum at a time. Bags and buckets of removed matrix and bone were carefully dried and later wet-screened through c. 17 mesh screens either onsite or at the Red Mountain Museum laboratory (Parmalee, 1992). Screened matrix was later picked for faunal remains. Individual elements that could be identified at least to the order level were cataloged individually with Red Mountain Museum (RMM) catalog numbers while the remaining bones from each unit/stratum were assigned bulk RMM catalog numbers. Large amounts of bulk samples (>100 kg) were taken from the cave for later processing. In 1994, the Red Mountain Museum merged with McWane Science Center, and the Bell Cave materials were transferred to their current repository in downtown Birmingham, AL. As a result, Bell Cave materials processed or cataloged after 1994 were given distinct McWane Science Center (MSC) catalog numbers. Red Mountain Museum personnel assigned Bell Cave the Alabama paleontological site acronym ACb-2, a unique designation used as part of a standard locality system employed by all paleontologists in the state of Alabama, USA. All of the materials from these excavation efforts are currently housed in the collections at McWane Science Center in Birmingham, Alabama (the permanent repository for the former Red Mountain Museum collections) and are available to qualified researchers for study.

In total, 235 cataloged lots were examined as part of this study. Each element examined was identified to lowest possible taxonomic level using available literature and comparative collections (see ‘Systematic Paleontology’ section below) and recorded in MS Excel. In the event a cataloged lot contained multiple identified taxa, the original catalog number was retained, but sub-numbers were assigned (RMM 6000.1, RMM 6000.2, etc.). Multiple elements associated with a single individual were given a single catalog number. All figured specimens were photographed with a tripod mounted Nikon d3000 camera with Nikkor 60 mm lens, and broken specimens were repaired (when possible) with B-76 butvar. Specimen photographs were rendered in Adobe Photoshop CS2 software as part of the production of the presented figures. Due to the number of specimens/fragments it was not feasible to photograph all materials. However, all lots and specimens are available for study upon request.

Bell Cave fish taxa identified as part of this study are provided in Appendices S1 and S2, and a complete list of all unequivocal taxa reported from all sites is provided in Table 1. All taxonomy presented in Tables, Appendices, and Systematic Sections follows Etnier and Starnes’ *The Fishes of Tennessee* (1993) and Boschung and Mayden’s *Fishes of Alabama* (2004).

**Table 1:** Late Pleistocene fish faunas from the Tennessee River Basin, Alabama, USA. Basin status is based on state records following *The Fishes of Tennessee* Etnier & Starnes (1993) and *Fishes of Alabama* Boschung & Mayden (2004). Mainstem survey data from gillnet and electrofishing survey data provided by TVA biologists and is specific to Tennessee River mainstem only. “Native” refers to taxa that have been present in the Tennessee River Basin since before historic times (Etnier & Starnes, 1993; Boschung & Mayden, 2004). “Not recorded” refers to taxa that have not previously been reported from the Tennessee River Basin (Etnier & Starnes, 1993; Boschung & Mayden, 2004).

Taxa	1: Bell Cave, ACb-2	2: ACb-3	3: ACb-5	4: Appalachian Caverns	5: Baker Bluff Cave	6: Beartown Cave	7: Cheek Bend Cave	8: Dust Cave	9: Guy Wilson Cave	10: Saltville	Contemporary Tennessee River Basin Status	Mainstem surveys
**Acipenseriformes**												
**Acipenseridae**								+				
*Acipenser*												
*A. fulvescens*	+						+				Native	+
*Scaphirhynchus*												
*S. platorynchus*	+										Native	
**Lepisosteiformes**												
**Lepisosteidae**												
*Lepisosteus*					+		+					
*L. osseus*	+										Native	+
**Anguilliformes**												
**Anguillidae**												
*Anguilla*												
*A. rostra*	+										Native	+
**Esociformes**												
**Esocidae**												
*Esox*					+							
*E. masquinongy*	+						+				Introduced	+
*E. lucius*	+										Introduced	
**Cypriniformes**												
**Cyprinidae**				+	+					+		
*Campostoma*	+						+				Native	+
*Luxilus*												
*L. cornutus*										+	Not recorded	
*Nocomis*									+			
*N. bigutattus*							+				Not recorded	
*N. micropogon*	+						+				Native	
*Pimephales*												
*P. notatus*										+	Native	+
*Semotilus*												
*S. atromaculatus*							+				Native	+
**Catostomidae**				+				+	+	+		
*Catostomus*												
*C. commersoni*	+				+		+			+	Native	
*Ictiobus*	+										Native	+
*Hypentelium*												
*H. nigricans*				+	+		+		+		Native	+
*Moxostoma*				+	+			+				
*M. anisurum*							+				Native	+
*M. carinatum*	+			+	+		+				Native	+
*M. duquesnei*					+		+				Native	+
*M. erythrurum*					+		+	+			Native	+
*M. lacerum*	+										Native/ extinct	
*M. macrolepidotum*	+						+				Native	
**Siluriformes**												
**Ictaluridae**		+						+				
*Ameiurus*	+										Native	+
*Ictalurus*					+	+						
*I. punctatus*	+				+			+			Native	+
*Noturus*		+			+							
*N. flavus*	+						+				Native	
*N. eleutherus*	+										Native	
*N. stigmosus*	+										Native	
*N. elegans*	+										Native	
*N. flavater*	+										Not recorded	
*Pylodictus*												
*P. olivaris*	+										Native	+
**Scorpaeniformes**												
**Cottidae**												
*Cottus*	+			+								
*C. carolinae*							+				Native	+
*C. bairdi*							+				Native	+
**Perciformes**												
**Centrarchidae**										+		
*Ambloplites*												
*A. rupestris*	+			+	+		+		+	+	Native	+
*Lepomis*								+				
*L. cyanellus*							+				Native	+
*Micropterus*		+			+							
*M. dolomieu*	+				+						Native	+
*M. punctatus*	+		+								Native	+
**Percidae**												
*Etheostoma*												
*E. blennioides*							+				Native	+
*Perca*												
*P. flavescens*							+				Native	+
*Percina*												
*P. caprodes*							+				Native	+
*Sander*					+			+				
*S. canadensis*	+				+		+				Native	+
*S. vitreus*	+						+				Native	+
**Sciaenidae**												
*Aplodinotus*												
*A. grunniens*	+	+			+		+	+			Native	+

### Bell cave site description

**Figure 2:**
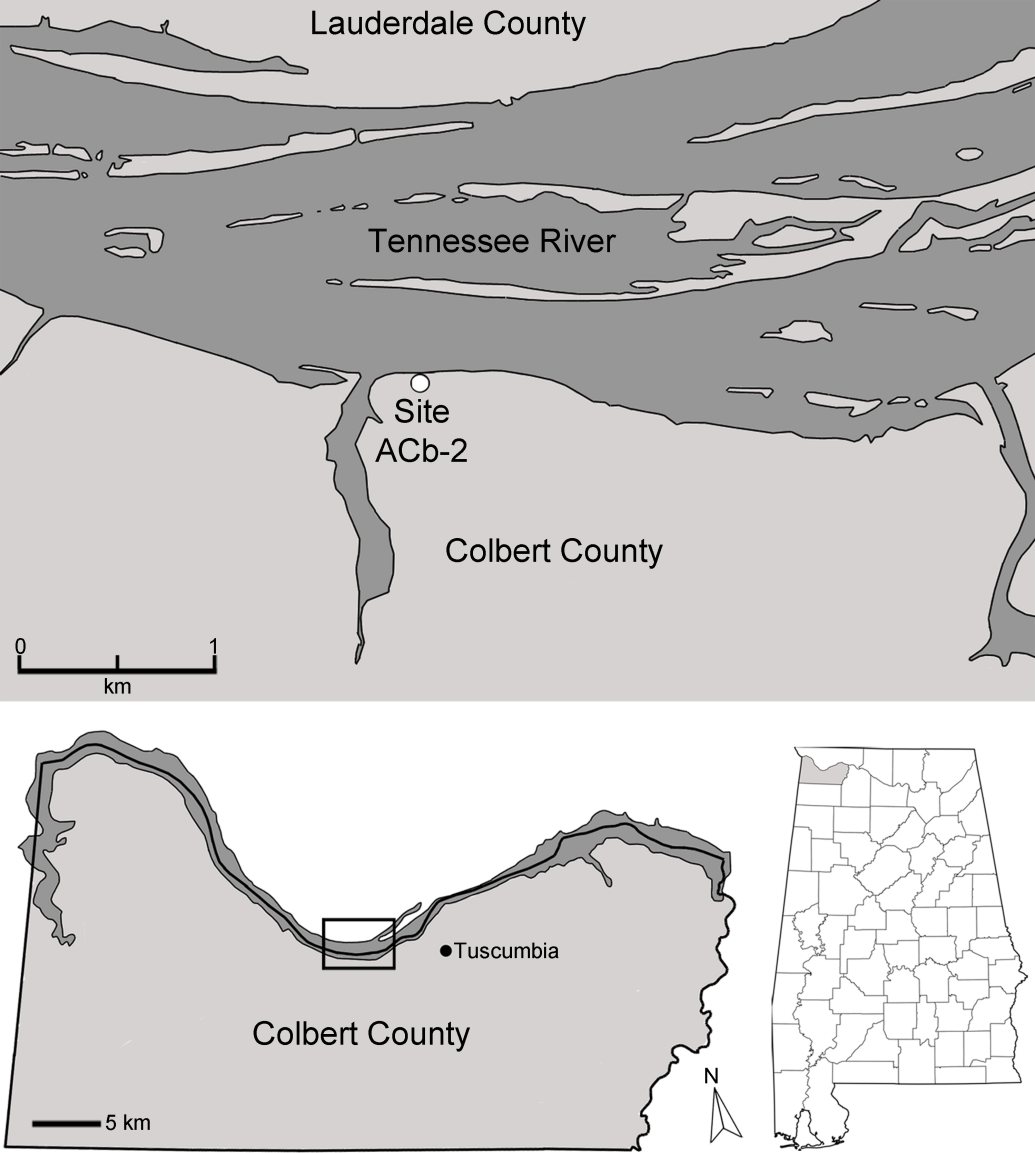
Map of the Tennessee River in Alabama, USA, and the location of Bell Cave (site ACb-2).

**Figure 3:**
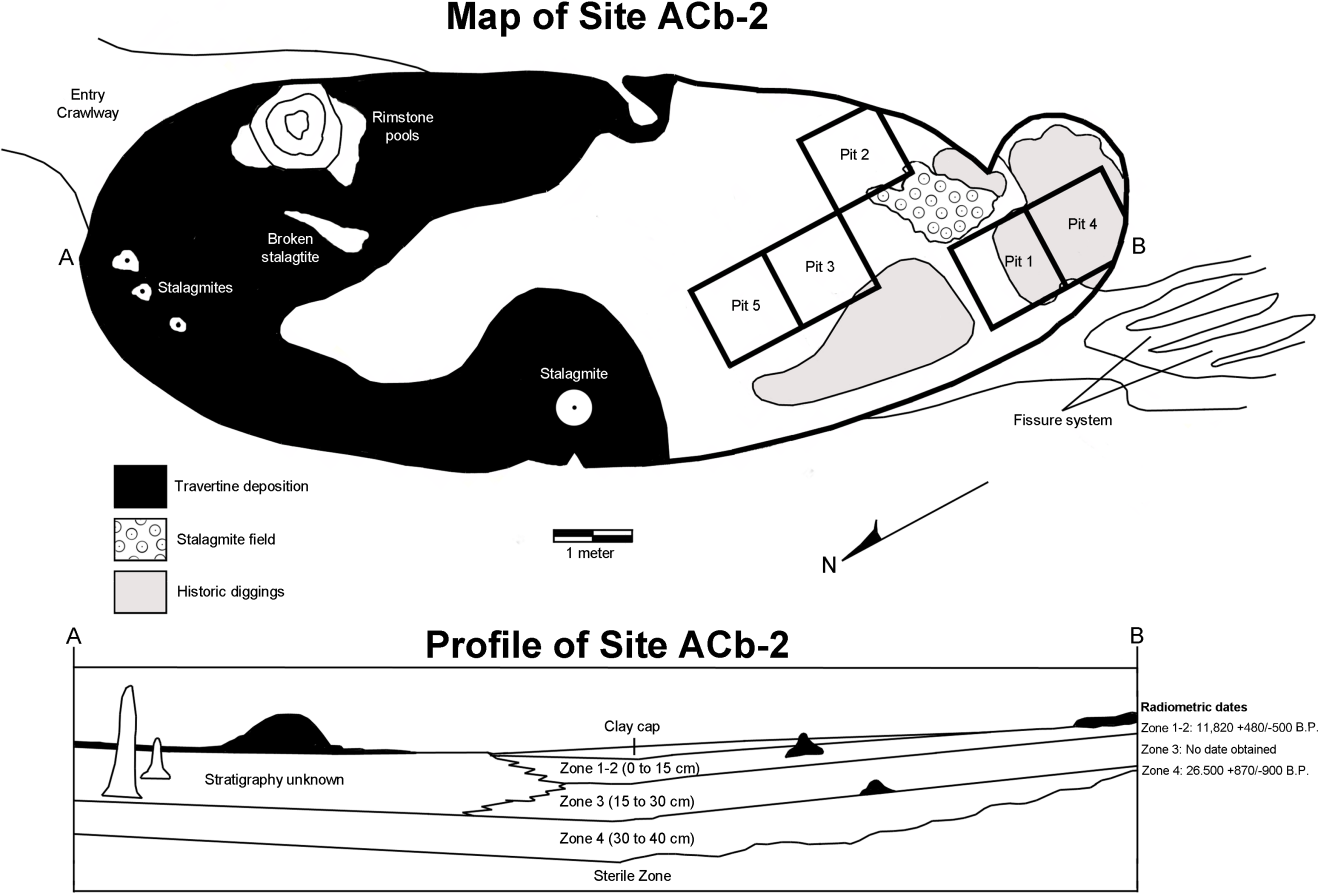
Map and stratigraphic profile of Bell Cave (site ACb-2), Colbert County, AL, USA.

Bell Cave is located along the south bank of the Tennessee River in the northwest corner of Alabama in Colbert County (Fig. 2). Situated approximately 11 km west of the town of Tuscumbia, the cave is located near Tennessee River Mile 248.2 at 87°47′32″N, 34°43′46″W (Churcher et al., 1989; see also Holman, Bell & Lamb, 1990; Martin & Prince, 1990; Parmalee, 1992). The cave formed within the middle Mississippian-age Tuscumbia Limestone and the modern entrance to the cave overlooks the Tennessee River and sits approximately 15 m above the current mean water level (Martin & Prince, 1990; Parmalee, 1992). The cave consists of a 26.2 m horizontal crawlway that opens into a large 4.0 by 11.9 m oblong “pit-like” room with a ceiling that does not exceed 10.5 m in height (Fig. 3; Churcher et al., 1989; Holman, Bell & Lamb, 1990). This oblong room is oriented northeast to southwest and has become a settling basin for fissure-transported sediments (Churcher et al., 1989; Holman, Bell & Lamb, 1990; Parmalee, 1992). The southwest wall of the cave reveals a floor-to-ceiling fissure that likely served as the primary entrance during the period of bone accumulation (Parmalee, 1992). On the floor in the southwestern half of the cave, evidence of historic digging is present (Fig. 3), likely the result of 19th century saltpeter mining.

Museum excavations within Bell Cave revealed an accumulation of approximately 70 cm of sterile clay overlain by 40 cm of bone packed mud (Fig. 3; Holman, Bell & Lamb, 1990; Martin & Prince, 1990; Parmalee, 1992). Vertebrate remains from the site were recovered from four lithologically distinct, stratigraphically-controlled levels, referred to herein as “zones” (Fig. 3; Bell, 1985; see also Holman, Bell & Lamb, 1990; Martin & Prince, 1990; Parmalee, 1992). These bone-containing zones each represent unique depositional events and can literally be “peeled” from one another (Bell, 1985; Holman, Bell & Lamb, 1990; Martin & Prince, 1990; Parmalee, 1992). The lowest 70 cm of the site represents a sterile layer and is overlain by the lowermost bone-bearing stratum, Zone 4 (Bell, 1985; Martin & Prince, 1990). These two layers consist of yellow laminated clays that were subaqueously deposited at a time when the water level of the Tennessee River was much higher than that of present day (Bell, 1985; Churcher et al., 1989). Desiccation cracks and settling at the top of Zone 4 indicate a period of dewatering and consolidation of these two layers and the onset of travertine formation at the top of Zone 4 (Bell, 1985). These travertine deposits suggest that some time had elapsed between the dewatering and the deposition of the upper three layers, Zones 1 through 3.

Zones 1, 2, and 3 consist of bone-filled reddish textured clays that were deposited as independent mud flow events from fissures at the southwest end and in the ceiling of the main cave room (Martin & Prince, 1990). Zones 1 and 2 were combined and referred to as Zone 1–2 since they could not be distinguished in certain parts of the cave (Parmalee, 1992). Bell (1985) and Churcher et al. (1989) reported the following radiocarbon dates from bone samples taken from the four bone-bearing zones:

**Zone 1–2 (0–15 cm)—**11,820 + 480/−500 B.P. Dated from an *Ursus americanus* femur collected in the upper part of Zone 2 and the lower part of Zone 1. Date determined by Dicard Radioisotope Company (Norman, OK)—sample DIC 2929, 1(222-02-3).

**Zone 3 (15–30 cm)—**Not enough large bone was present for dating, but this zone has a similar sedimentary profile and is faunistically identical to Zone 1–2.

**Zone 4 (30–40 cm)—**26,500 + 870/−990 B.P. Dated from large longbone fragments collected at the base of Zone 4, just above the sterile layer. Date determined by Dicard Radioisotope Company (Norman, OK)—sample DIC 3117, 236-61. As a specific caveat to prior literature on the dating of these zones, in a study of the Bell Cave herpetofauna, Holman (1995) failed to report the presence of Zone 4 and incorrectly assigned the date of 26,500 B.P. to Zone 3.

Overall, Zones 1–2 and 3 represent secondary depositional events in which consolidation and mixing within each zone may have occurred (but not between them; Martin & Prince, 1990). Although the main sources of bone accumulation within the cave were the result of subaqueous deposition (Zone 4) and viscous mudflows (Zones 1–2 and 3), other methods of bone accumulation were also evident. Some bone material likely fell or “filtered” through a former fissure located above the cave as well as death during denning or hibernation; predation by predators also likely contributed to the accumulation (Churcher et al., 1989; Parmalee, 1992). The former fissure at the southwest end of the cave would have been large enough to serve as an entrance for large carnivores such as wolves and bears (which are among the mammalian remains recovered; see Ebersole & Ebersole, 2011) and the presence of raptor remains suggest the possibility of roosting within the cave (Parmalee, 1992). Other recovered specimens show excessive gnawing produced by rodents and other scavengers (Holman, Bell & Lamb, 1990), suggesting the possibility of rodent transport of some material. Human remains were recovered from clay and travertine cap deposits located above Zone 1–2. The discovery of no such remains from any of the underlying zones suggests the 40 cm of bone accumulation occurred before any significant human populations were in the area (Bell, 1985).

## Results

The 10 late Pleistocene localities examined within the Tennessee River Basin (Fig. 1) produced 41 unequivocal taxa (Table 1). These taxa are represented by 8 orders, 11 families, 27 genera, and 38 species of freshwater fish. The most species rich site was the fauna from Bell Cave (Acb-2) which is noted in a separate systematic paleontology discussion below that includes specific notes on the recovered 11 families, 19 genera, and 24 identifiable species (28 unequivocal taxa) from this locale with discussions of these families in the context of all known late Pleistocene sites from the Tennessee River Basin.

### Systematic paleontology

Referred Material—see Appendix S1 for specific lot catalog numbers, type and quantity of specific elements, and stratigraphic zone information (arranged as Zones 1–2, 3, 4, and Mixed). Mixed areas are ‘disturbed areas’ and refer to any specimen in which a specific zone could not be determined, such as those from superficial caps, within fissures, resultant of historic diggings, or from rimstone areas within the cave deposits (Fig. 3; see Appendix S1 for specific position in the cave). All identified material referenced the University of Tennessee Zooarchaeology Collections (Knoxville, TN, USA) or comparative skeletal material from the personal collection of WC Dickinson. All taxonomy and references to biogeography, ecology, conservation status, and Tennessee River Basin occurrences referenced Etnier and Starnes’ *The Fishes of Tennessee* (1993), Boschung and Mayden’s *Fishes of Alabama* (2004), and Page and Burr’s *Field Guide to Freshwater Fishes* (2011). References to current mainstem taxa occurrences and abundances follow recent (1993–2014) Tennessee River mainstem electrofishing and gillnet surveys undertaken by Tennessee Valley Authority (TVA) Biologists (Kurt Lakin, TVA Biologist, personal communication). The systematic arrangement of higher taxonomic rankings follows that of Caroll (1988).

**Table:** 

CLASS OSTEICHTHYES Huxley, 1880

Material

Fin rays, vertebrae, dentaries, premaxillae, teeth, operculi, spines, unknown fragments.

Occurrences

Zones 1–2, 3, 4, Mixed.

Remarks

Although much of the Bell Cave material could be identified at least to the family level (450 unique identifications arranged in lots), 114 collections of material still remain problematic for definitive identification. The majority of this material is comprised of unidentifiable vertebrae and fin rays; however, several other fragments carry notes in Appendix S1 as to suspected identifications and potential significance. The majority of these unidentified fragments almost certainly come from the taxa outlined below—however, several fragments may amend new taxa to the list. For example, lot RMM 4544 contains the unidentified remains of a dentate fragment with several tiny recurved teeth *in situ*. This element could represent a possible Hiodontidae member. Additionally, lot RMM 4950 contains a few cranial elements that could represent a single Bowfin *Amia calva* (Amiidae) individual. Other unidentified fragments may actually be smaller non-fish vertebrate fragments such as mammal or bird remains mistakenly apportioned with fishes. Other fragments may be too eroded or amorphic for any identification attempts and remain cataloged as such. Regardless, additional matrix from future cave excavations may result in further taxonomic detail and resolution.

A caveat to any discussion of late Pleistocene fish remains is that these collections almost undoubtedly represent a subset of the actual living assemblage and that numerous taxa are likely missing due to some form of taphonomic bias occurring between decomposition, transport, deposition, preservation, and collection processing. For example, the complete absence of otoliths among the examined material or within the >100 kg of recovered bulk matrix from Bell Cave leads us to believe this absence is preservational. The chemical composition of nearly all Actinopterygiian otoliths is aragonite (as opposed to bone and teeth which are composed of apatite; Hall & Kennedy, 1967), a form of calcium carbonate that is often subject to erosion by synsedimentary or postsedimentary decalcification when in the presence of infiltrating acidic water (Nolf, 1985). The former presence of acidic water within Bell Cave is evident by not only the formation of the limestone cave itself, but by the geomorphological events that formed the various strata (subaqueous and mud-flow deposition) and cave formations (i.e., travertine and speleothems). Additionally, the likely underrepresentation of smaller taxa (e.g., Minnows and Darters) may represent a combination of factors ranging from deposition (lighter fragments may be less readily held in deposits), preservation (smaller bone fragments weather more rapidly than larger fragments), decomposition (may degrade more rapidly than larger fragments, particularly in digestive tracts of larger animals such as owls), and/or collection processing (screen mesh size may exclude the tiniest fragments) biases. These taphonomic biases are also evident within the nine other localities discussed in this study, as all report fish taxa that were not able to be identified at or below the order level.

**Table:** 

ORDER ACIPENSERIFORMES Berg, 1940
FAMILY ACIPENSERIDAE Linnaeus, 1758
*ACIPENSER FULVESCENS* OR *SCAPHIRHYNCHUS PLATORYNCHUS*
SHOVELNOSE STURGEON OR LAKE STURGEON


Material

Fulcrum, scute, lateral plate, quadrate, unidentified skull fragments.

Occurrences

Zones 1–2, 3, 4, Mixed

**Table:** 

**Figure 4:**
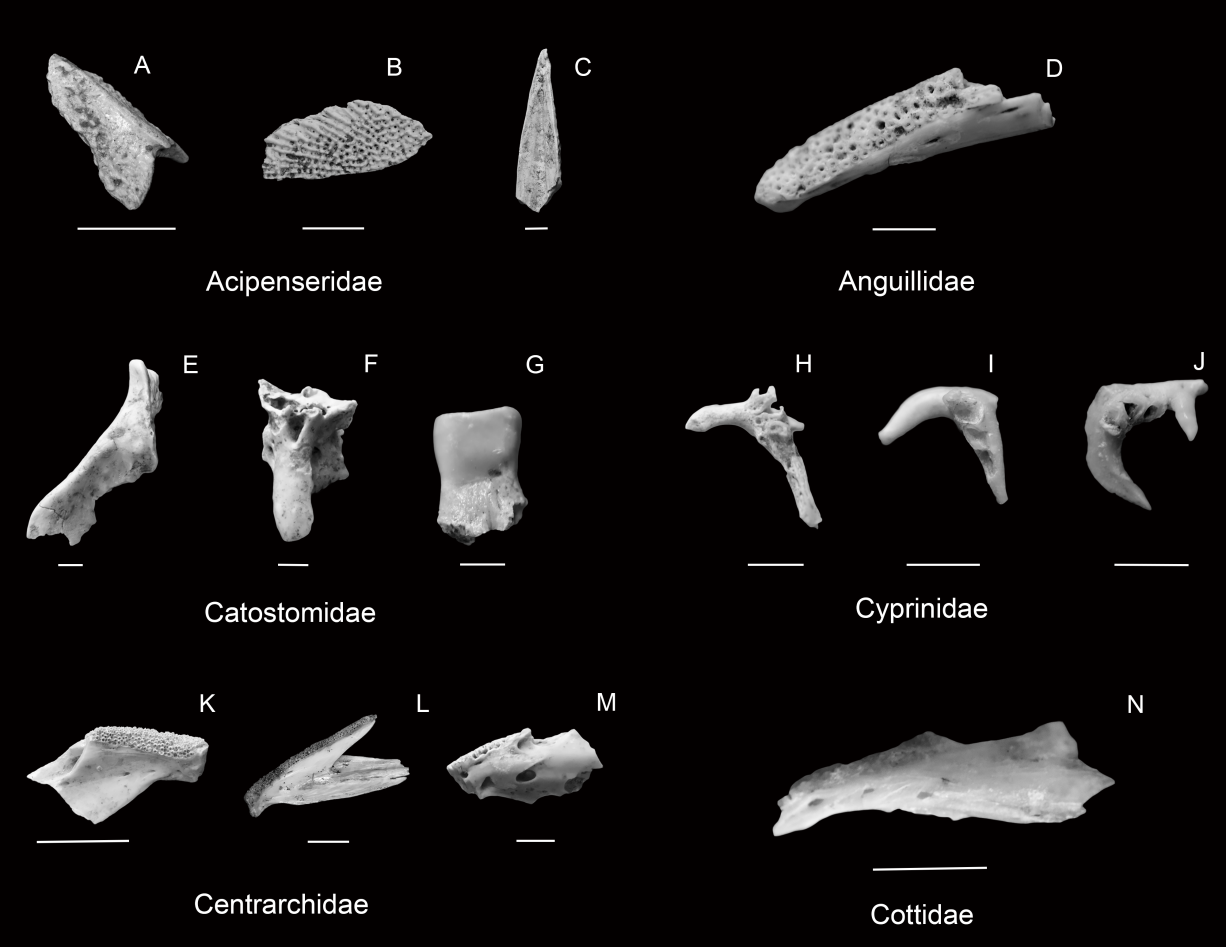
Selected diagnostic elements from Bell Cave samples: Acipenseridae ((A) lateral view of sturgeon *Acipenser* sp. scute from dorsal median row (scale 1 cm; RMM 3900.1), (B) lateral view of sturgeon *Acipenser* sp. scute from lateral row (scale 1 cm; RMM 4979.1), (C) lateral view of sturgeon *Acipenser fulvescens* fulcrum (scale 2 mm RMM 4665.1)), Anguillidae ((D) mediodorsal view of right eel *Anguilla rostrata* dentary (scale 2 mm; RMM 5268.1)), Catostomidae ((E) lateral view of harelip sucker *Moxostoma lacerum* premaxilla (scale 2 mm; RMM 5265.5), (F) lateral view of harelip sucker *Moxostoma lacerum* palatine (scale 2 mm; RMM 5265.5), (G) medial view of *M. carinatum* pharyngeal tooth (scale 2 mm; RMM 4793.1)), Cyprinidae ((H) medial view of Cyprinidae right pharyngeal (scale 2 mm; RMM 5175.7), (I) medial view of *Nocomis* sp. right pharyngeal (scale 2 mm; RMM 4181.5), (J) medial view of *Nocomis* sp. right pharyngeal (scale 2 mm; RMM 4544.3)), Centrarchidae ((K) anterior portion of *Micropterus dolomieu* left dentary (scale 1 cm; RMM 3900.6), (L) complete *M. punctatus* right dentary (scale 1 cm; RMM 6651.1), (M) anterior portion of *Ambloplites rupestris* left dentary (scale 2 mm; RMM 5268.3)), Cottidae ((N) lateral view of *Cottus* sp. left dentary (scale 2 mm; RMM 5268.5)).

GENUS *ACIPENSER* Linnaeus, 1758
*ACIPENSER FULVESCENS* Rafinesque, 1817
LAKE STURGEON
Figures 4A–4C


Material

Fulcrum, scute, parasphenoid.

**Figure 5:**
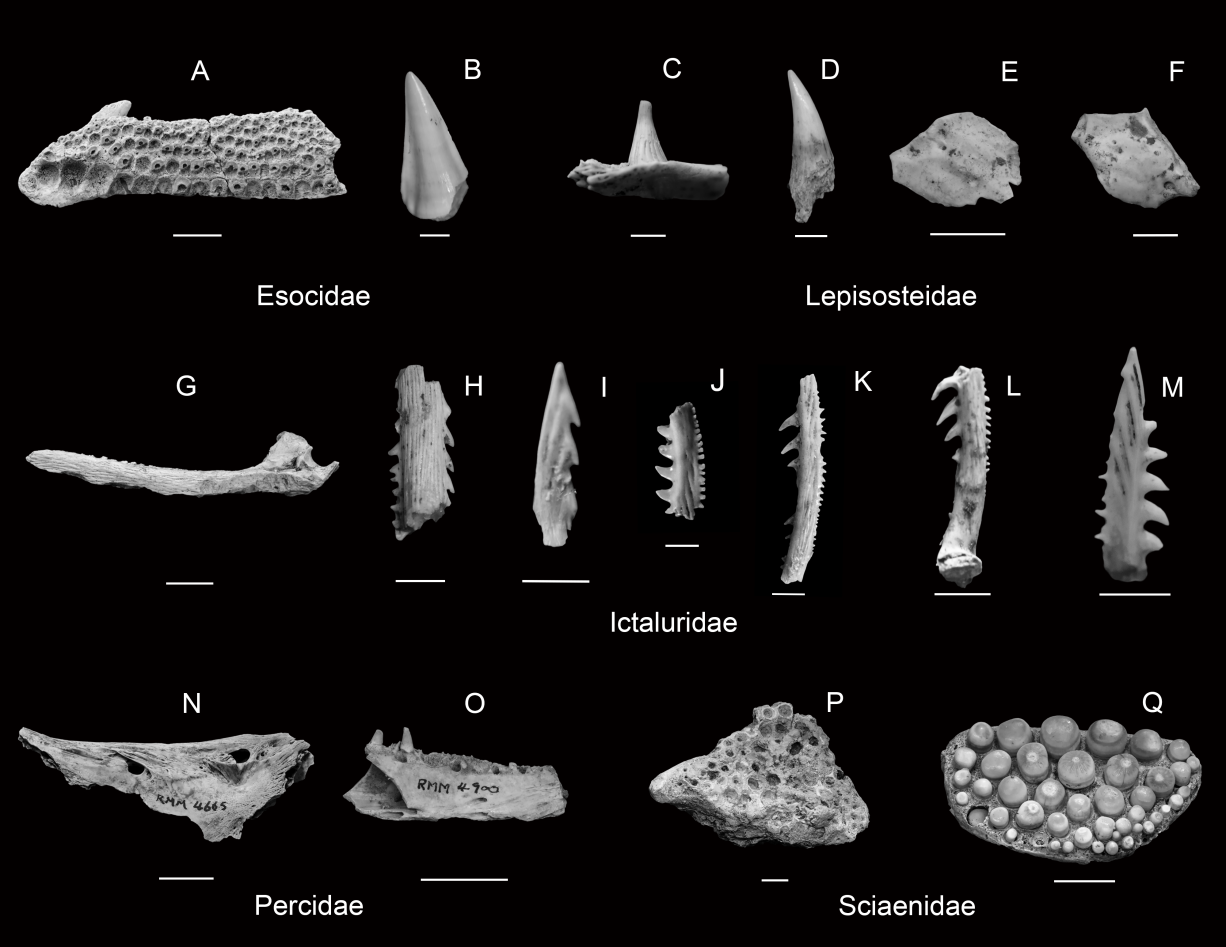
Selected diagnostic elements from Esocidae ((A) ventral view of *Esox masquinongy* left palatine (scale 1 cm; RMM 4675.1), (B) lateral view of *E. lucius* tooth (scale 2 mm; RMM 4093.2)), Lepisosteidae ((C) lateral view of *Lepisosteus* sp. tooth *in situ* (scale 2 mm; RMM 5273.1), (D) lateral view of *Lepisosteus sp.* tooth fragment (scale 2 mm; RMM 3796.4), (E) lateral view of *Lepisosteus* sp. ganoid scale (scale 1 cm; RMM 4032.1), (F) lateral view of *Lepisosteus* sp. ganoid scale (scale 1 cm; RMM 4660.2)), Ictaluridae ((G) right *Ictalurus* sp. pectoral spine (scale 1 cm; RMM 3794.2), (H) *Pylodictus olivaris* pectoral spine fragment (scale 2 mm; RMM 4182.2), (I) *Noturus* sp. cf. *N. flavus* pectoral spine fragment (scale 2 mm; RMM 5177.6), (J) *Noturus* sp. cf. *N. flavater* pectoral spine fragment (scale 2 mm; RMM 4944.3), (K) *Noturus* sp. cf. *N. elegans* pectoral spine fragment (scale 2 mm; RMM 4898.3), (L) *N. eleutherus* left pectoral spine fragment (scale 2 mm; RMM 3794.4), (M) *N. stigmosus* pectoral spine fragment (scale 2 mm; RMM 4231.2)), Percidae ((N) mesial view of *Sander* sp. left dentary (scale 1 cm; RMM 4665.10), (O) mesial view of *Sander* sp. left dentary (scale 1 cm; RMM 4900.4)), Sciaenidae ((P) *Aplodinotus grunniens* infrapharyngeal fragment (scale 1 cm; RMM 5224.1), (Q) *A. grunniens* suprapharyngeal with teeth (scale 1 cm; RMM 4127.1)).

Occurrences

Zone 3.

**Table:** 

GENUS *SCAPHIRHYNCHUS* Heckel, 1835
*SCAPHIRHYNCHUS PLATORYNCHUS* (Rafinesque, 1820)
SHOVELNOSE STURGEON

Material

Scute.

Occurrences

Mixed.

Remarks

The presence of these remains confirms the native status of these taxa to the Tennessee River drainage. However, a combination of harvest and habitat alterations have resulted in the depletion of both of these taxa throughout the modern Tennessee River Basin (Etnier & Starnes, 1993; Boschung & Mayden, 2004; Appendix S2). Specifically, Boschung & Mayden (2004) list this family as entirely extirpated from the basin; however, recent TVA mainstem fisheries surveys did recover a small number of Lake Sturgeon (8 out of 687,334 total collected fish). The presence of Lake Sturgeon in recent surveys is unlikely an error in Boschung & Mayden (2004); rather, their presence is almost undoubtedly the result of recent stocking efforts to reestablish breeding populations in portions of the Lake Sturgeon’s native range. Scutes, plates, and fragmentary material were definitively identified on the basis of appearance and thickness. Reference to recent comparative material facilitated some differentiation of a few fragments given robustness and thickness differences as well as sheer size to distinguish between *Acipenser* and *Scaphirhynchus* material. In addition to the Bell Cave specimens, reports of the presence of Acipenseriformes remains have come from Dust Cave in Alabama (Walker, 1998), and more specifically, Lake Sturgeon, from Cheek Bend Cave in Tennessee (Dickinson, 1986). Interestingly, Bell Cave is the only locality within our study area to contain late Pleistocene remains of the Shovelnose Sturgeon (see Table 1).

**Table:** 

ORDER LEPISOSTEIFORMES Hay, 1926
FAMILY LEPISOSTEIDAE Cuvier, 1825
GENUS *LEPISOSTEUS* Lacépède, 1803
*LEPISOSTEUS* SP.
GAR
Figures 5C–5F

Material

Teeth, scales, dentary, vertebrae, skull fragments.

Occurrences

Zones 1–2, 3, 4, Mixed.

**Table:** 

*LEPISOSTEUS* SP. CF. *L. OSSEUS* (Linnaeus, 1758)
LONGNOSE GAR

Material

Dentary, teeth.

Occurrences

Zones 1–2, 3.

Remarks

Four Lepisosteidae taxa are recognized as native to the Tennessee River drainage. Recent mainstem surveys have confirmed the presence of these taxa: Longnose Gar (*Lepisosteus osseus*), Spotted Gar (*L. oculatus*), and Shortnose Gar (*L. platostomus*), once considered extirpated from this section of the basin (Boschung & Mayden, 2004). However, modern surveys have not collected any Alligator Gar (*Atractosteus spatula*). Together, the three *Lepisosteus* taxa comprise 1,809 recorded individuals, with Spotted Gar representing the most common. In the Bell Cave samples, their characteristic ganoid scales coated with ganoin enamel were the most numerous elements identified from Gar taxa. The presence of fine needle-like striated teeth of varying position rooted with a circular base in a shallow socket were also used to delineate Lepisosteidae. Only one dentary element associated with the recovered material was diagnostic beyond the generic level. This particularly long and narrow dentary bone with teeth *in situ* strongly suggests the presence of Longnose Gar in the samples, while conversely, the presence of more compressed dentary bones with teeth *in situ* likely indicate the presence of Spotted and/or Shortnose Gar. Several extremely large Lepisosteidae fragments, including comparatively thick scales, may also indicate the presence of Alligator Gar in the Bell Cave samples. However, no Alligator Gar are known to have ever been collected from the mainstem Tennessee and there is not enough intact diagnostic material to confirm this taxon. In addition to the Bell Cave specimens, remains identified as *Lepisosteus* sp. have been identified from Baker Bluff (Guilday et al., 1978) and Cheek Bend (Dickinson, 1986) caves in Tennessee (see Table 1).

**Table:** 

ORDER ANGUILLIFORMES Greenwood et al., 1966
FAMILY ANGUILLIDAE Garsault, 1764
GENUS *ANGUILLA* Schrank, 1798
*ANGUILLA ROSTRATA* (Lesueur, 1817a)
AMERICAN EEL
Figure 4D

Material

Dentary, premaxillary.

Occurrences

Zone 3.

Remarks

The American Eel is an uncommon native catadromous taxon present in large rivers throughout the modern Mississippi River drainage, including the Tennessee River. Specific to the mainstem, only ten individuals were collected in recent TVA surveys that were composed of 687,334 total individuals. The American Eel depends on long migration runs between spawning areas in the north Atlantic and streams throughout the Mississippi River drainage to fulfill its life cycle, this highly migratory attribute has contributed to its decline in recent years as a result of impoundments and river fragmentation (Etnier & Starnes, 1993; Boschung & Mayden, 2004). Definitive remains in the Bell Cave samples were initially flagged as unique considering the elongated structure of the dentary and premaxillary bones while identification was confirmed through corresponding tooth socket shape and offset arrangement. None of the samples yielded any evidence of the tiny recurved teeth from these socket positions. No additional Anguilliformes have been reported from any other known late Pleistocene locality within our study area (see Table 1).

**Table:** 

ORDER ESOCIFORMES Johnson & Patterson, 1996
FAMILY ESOCIDAE Berg, 1940
GENUS *ESOX*Linnaeus, 1758
*ESOX* SP. CF. *E. LUCIUS* Linnaeus, 1758
NORTHERN PIKE
Figure 5B

Material

Teeth.

Occurrences

Zones 3.

**Table:** 

*ESOX MASQUINONGY* Mitchill, 1824
MUSKELLUNGE
Figure 5A

Material

Teeth, vomer, palatine, angular, dentary.

Occurrences

Zones 1–2, 3, 4.

**Table:** 

*ESOX* SP.
*ESOX LUCIUS* OR *ESOX MASQUINONGY*
NORTHERN PIKE OR MUSKELLUNGE

Material

Teeth, vertebrae, angular, palatine, vomer, dentary.

Occurrences

Zones 1–2, 3, 4, Mixed.

Remarks

The presence of these remains indicate a range extension into Alabama for two of these taxa. Boschung & Mayden (2004) indicate that only two species of Esocidae are native to the Tennessee River drainage, the Chain Pickerel (*Esox niger*) and the Redfin Pickeral (*E. americanus*), while Etnier & Starnes (1993) add the Muskellunge to this list. Recent TVA fisheries surveys of the mainstem did result in the collection of Chain Pickerel, Redfin Pickerel, as well as Muskellunge. Interestingly, the presence of Northern Pike in Bell Cave samples coupled with the absence in modern fisheries surveys or listed as native in species accounts likely indicates a Pleistocene displacement event. Page & Burr (2011) indicate that the Northern Pike’s range encompasses only the northern portion of the Mississippi River, Great Lakes, Arctic, and Pacific basins while Muskellunge occupy a native range (irrespective of introduced populations) that also encompasses the northern portion of the Mississippi River Basin and the southern portion of the Great Lakes Basin.

Several teeth, vertebrae, skull fragments, and a single dentary were definitively identified on the basis of meristic and morphometric appearance and size. Crossman & Harrington (1970) point out that the teeth of Northern Pike exhibit compressed lateral edges on the larger teeth, a feature absent in Muskellunge. However, in this same publication, it is mentioned that these compressed edges are not always present, thus, the tentative assignment of the majority of the unassociated Bell Cave *Esox* tooth samples. The large size of the teeth were used to rule out either the ‘native’ Redfin or Chain Pickerel. One example of a large, intact palatine with tooth socket combinations was also used to definitively identify the presence of Muskellunge. When present, neural arches were also used to distinguish between Northern Pike and Muskellunge vertebrae. A near complete Muskellunge dentary was identified on the basis of submandibular pore counts (6–9 *E. masquinongy*, ∼4 *E. lucius*; Crossman & Harrington, 1970). In addition to the Bell Cave specimens, remains of *Esox* sp. have been reported from Baker Bluff Cave in Tennessee (Guilday, Hamilton & Parmalee, 1975), and remains of Muskellunge have been reported from Cheek Bend Cave in Tennessee (Dickinson, 1986). Bell Cave is the only locality within our study area to produce identifiable remains of the Northern Pike (see Table 1).

**Table:** 

ORDER CYPRINIFORMES Bleeker, 1859
FAMILY CYPRINIDAE Cuvier, 1817
MINNOWS
Figure 5H

Material

Pharyngeal, pharyngeal teeth, vertebrae.

Occurrences

Zones 1–2, 3, 4, Mixed.

**Table:** 

GENUS *CAMPOSTOMA* Agassiz, 1855
*CAMPOSTOMA* SP.
STONEROLLER

Material

Pharyngeal.

Occurrences

Zones 1–2.

**Table:** 

GENUS *NOCOMIS* Girard, 1856
*NOCOMIS* SP.
‘RIVER’ CHUBS
Figs. 4I–4J

Material

Pharyngeal.

Occurrences

Zones 1–2.

**Table:** 

*NOCOMIS* SP. CF. *N. MICROPOGON* (Cope, 1865)
RIVER CHUB

Material

Pharyngeal.

Occurrences

Zones 1–2.

Remarks

Etnier & Starnes (1993) and Boschung & Mayden (2004) recognize 91 species of native Tennessee River Basin Cyprinidae, while recent surveys of the mainstem indicate the presence of 21 of these native taxa. The Largescale Stoneroller (*Campostoma oligolepis*), and Central Stoneroller (*C. anomalum*), were identified in recent mainstem surveys in Alabama; however, only the Central Stoneroller is considered native to the basin by Etnier & Starnes (1993). The Largescale Stoneroller could be a recent introduction or represent the recently described native Bluefin Stoneroller (*C. pauciradii*). However, the Bluefin Stoneroller is typically only associated with the Georgia portion of the Tennessee River Drainage Basin, well outside the vicinity of Bell Cave (Page & Burr, 2011). Regardless, the contemporary presence of these two Stoneroller taxa potentially narrow the identification of the Pleistocene remains. Specific to the River Chub group, *Nocomis* remains provide confirmation of a native taxon that has probably been extirpated from the mainstem. Two *Nocomis* taxa, the Bluehead Chub (*N. leptocephalus*) and River Chub, are noted to occur in the basin, although the former is not always noted as present in the drainage basin (Boschung & Mayden, 2004). Overall, mainstem extirpation or population changes are likely a function of habitat alterations in the past century as well as the potential for gear bias to under-represent this and other Cyprinidae groups. Cyprinidae material was identified using pharyngeal arch shape and tooth formula (Eastman & Underhill, 1973; Boschung & Mayden, 2004). Comparative material of several Minnow genera, including *Erimystax* and *Cyprinella*, closely matched some of the noted tooth configurations; however, too much degradation prevented anything more than tentative identification. The overall lack of Cyprinidae remains could represent a change in assemblage, preservation bias, or collection and processing bias.

Late Pleistocene specimens identified as Cyprinidae have also been identified from Appalachian Caverns (this study) and Baker Bluff (Guilday et al., 1978) in Tennessee, and Saltville in Virginia (Dickinson, 1986). In addition to the specimen identified from Bell Cave, Stoneroller remains identified as *Campostoma* sp. have also been reported from Cheek Bend Cave in Tennessee (Dickinson, 1986). Remains belonging to ‘Chubs’ *Nocomis* sp. have also been identified from Guy Wilson Cave in Tennessee (this study), and although remains belonging to the River Chub have been identified from both Bell Cave and Cheek Bend Cave, the latter locality also produced remains belonging to Hornyhead Chub *N. biguttatus* (Dickinson, 1986). Other Cyprinidae members not identified from the Bell Cave deposits include Common Shiner *Luxilus cornutus* from Saltville in Virginia (Dickinson, 1986); Bluntnose Minnow *Pimephales notatus* from Saltville, and Creek Chub *Semotilus atromaculatus* from Cheek Bend Cave (Dickinson, 1986).

**Table:** 

FAMILY CATOSTOMIDAE Cope, 1871
SUCKERS

Material

Epihyal, hypohyal, basipterygium, hyomandibular, basihyal, scapula, pharyngeal teeth, supracleithrum, fin ray, quadrate, cleithrum, vertebrae, operculum, maxilla, orbitosphenoid, parietal, basioccipital, pharyngeal, angular, ethmoid.

Occurrences

Zones 1–2, 3, 4, Mixed.

**Table:** 

GENUS *CATOSTOMUS* Lesueur, 1817b
*CATOSTOMUS COMMERSONI* (Lacépède, 1803)
WHITE SUCKER

Material

Dentary.

Occurrences

Zones 1–2, 3.

**Table:** 

GENUS *ICTIOBUS* (Rafinesque, 1820)
*ICTIOBUS* SP.
BUFFALO

Material

Operculum, hyomandibular.

Occurrences

Zone 3.

**Table:** 

GENUS *MOXOSTOMA* (Rafinesque, 1820)
*MOXOSTOMA* SP.
REDHORSE

Material

Angular, operculum, rostral, epihyal, angular, parasphenoid, pharyngeal.

Occurrences

Zones 1–2, 3.

**Table:** 

*MOXOSTOMA CARINATUM* (Cope, 1870)
RIVER REDHORSE
Figure 4G

Material

Pharyngeal teeth, rostral, cleithrum, epihyal, maxillary, dentary, neural process, basioccipital, parietal, orbitosphenoid, scapula, urohyal.

Occurrences

Zones 1–2, 3, 4, Mixed.

**Table:** 

*MOXOSTOMA LACERUM* (Jordan & Brayton, 1877)
HARELIP SUCKER (*EXTINCT*)
Figures 4E–4F

Material

Premaxillary, palatine.

Occurrences

Zone 3.

**Table:** 

*MOXOSTOMA MACROLEPIDOTUM* (Lesueur, 1817b)
SHORTHEAD REDHORSE

Material

Dentary, maxillary.

Occurrences

Zones 1–2, 3.

Remarks

There are 22 species of North American Suckers native to the Tennessee River Basin (Etnier & Starnes, 1993; Boschung & Mayden, 2004), of which 14 are still collected in mainstem TVA surveys. The Bell Cave samples provide generic level evidence for all but the Spotted Sucker (*Minytrema melanops*), Northern Hogsucker (*Hypentelium nigricans*), and the Blue Sucker (*Cycleptus elongatus*). Genera represented include *Moxostoma*, *Ictiobus*, and *Catostomus*. All definitive species identified still occur in the mainstem, with one notable exception. Represented by only two cranial fragments, the remains of the extinct Harelip Sucker, are perhaps the most significant among the Bell Cave Catostomidae remains. Little is known about the Harelip Sucker’s niche, distribution, or eventual decline, except that the species is thought to have preferred medium to large warm-water lotic systems with low rates of sedimentation and that population declines occurred concurrently with these habitats becoming increasingly degraded as a result of agricultural sedimentation (Jenkins & Burkhead, 1994). The last living Harelip Sucker specimens were collected in 1893 in the Auglaize and Blanchard Rivers in Ohio, where previously collected individuals were distributed across portions of the Ohio River Basin and Great Lakes Basin (Jenkins & Burkhead, 1994). Manzano & Dickinson (1991) documented the first archaeological occurrences of the Harelip Sucker from several sites across Tennessee. Specimens from additional archaeological sites along the Tennessee River system in Tennessee and Alabama have been identified in recent years (WC Dickinson, 2015, unpublished data). The most diagnostic element found in the Bell Cave samples was the premaxillary bone, which can be described as having an obtuse angle approximately 140 degrees compared with more 90 degree premaxillary angles found in other Catostomidae (Manzano & Dickinson, 1991). The Harelip Sucker specimens identified from Bell Cave represent the first known Pleistocene occurrence for this taxon as well as the stratigraphically oldest occurrence recorded.

Other Catostomidae identifications were based on the morphology of dentaries, pharyngeal teeth, and operculi. Material from unidentified Redhorse is likely from one of the other identified Redhorse taxa. However, at least one example (RMM 5265) of a damaged or weathered cleithrum may be from a Silver Redhorse *M. anisurum*. Additionally, numerous damaged Catostomidae fragments may also be from one of three Carpsucker *Carpoides* taxa native to, and still collected in, the mainstem.

Within our study area, remains identified as belonging to Catostomidae have been identified from Dust Cave in Alabama (Walker, 1998), Appalachian Caverns (this study) and Guy Wilson Cave (this study) in Tennessee, and Saltville in Virginia (McDonald & Bartlett, 1983; Dickinson, 1986). The White Sucker has been reported from three localities aside from Bell Cave, including Baker Bluff (Guilday et al., 1978) and Cheek Bend caves in Tennessee (Dickinson, 1986), and Saltville in Virginia (Dickinson, 1986). Members of the Redhorse genus *Moxostoma* are widely represented within sites in our study area with specimens identified as *Moxostoma* sp. reported from Dust Cave, Appalachian Caverns, Baker Bluff Cave. Other Redhorse species found in Bell Cave have also been reported at other localities including River Redhorse from Appalachian Caverns, Baker Bluff Cave, and Cheek Bend Cave as well as Shorthead Redhorse from Cheek Bend Cave. Cheek Bend Cave has also produced three species of Redhorse not identified from Bell Cave, Silver Redhorse, Black Redhorse *M. dusquesnei* (also reported from Appalachian Caverns), and Golden Redhorse *M. erythrurum* (also reported from Dust Cave). Appalachian Caverns, Baker Bluff, Cheek Bend, and Guy Wilson caves also report the presence of Northern Hogsucker *Hypentellum nigricans*, another taxon not identified from Bell Cave. Of the localities within our study area, remains belonging to Buffalo are only known from Bell Cave (this study; see Table 1).

**Table:** 

ORDER SILURIFORMES Cuvier, 1817
FAMILY ICTALURIDAE Gill, 1861
NORTH AMERICAN CATFISH

Material

Pectoral spine, cleithrum, maxillary, dorsal spine, quadrate, symplectic, ceratohyal, palatine, premaxillary, coracoid, lateral ethmoid, posttemporal.

Occurrences

Zones 1–2, 3, 4, Mixed.

**Table:** 

GENUS *AMEIURUS* (Jordan & Evermann, 1896) *AMEIURUS* SP.
BULLHEAD CATFISH

Material

Dentary, spine.

Occurrences

Zones 1–2.

**Table:** 

GENUS *ICTALURUS* (Rafinesque, 1820)
*ICTALURUS* SP.
*ICTALURUS PUNCTATUS* OR *ICTALURUS FURCATUS*
CHANNEL CATFISH OR BLUE CATFISH
Figure 5G

Material

Dentary, pectoral spine, operculum, cleithrum, angular, premaxillary, quadrate, fin ray, hyomandibular, epihyal, ethmoid.

Occurrences

Zones 1–2, 3, 4, Mixed.

**Table:** 

*ICTALURUS PUNCTATUS* Rafinesque, 1818
CHANNEL CATFISH

Material

Pectoral spine, premaxilla, dentary, posttemporal, angular, hyomandibular, supraethmoid, cleithrum, maxillary.

Occurrences

Zones 1–2, 3, 4.

**Table:** 

GENUS *NOTURUS* Rafinesque, 1818
*NOTURUS* SP.
(*NOTURUS* SP. *[RABIDA]* or *NOTURUS* SP. *[SCHILBEODES]*)
MADTOM

Material

Pectoral spine, dentary.

Occurrences

Zones 1–2, 3, Mixed.

**Table:** 

*NOTURUS FLAVUS* (Rafinesque, 1818)
*NOTURUS* SP. CF. *N*. *FLAVUS* Rafinesque, 1818
STONECAT MADTOM
Figure 5I

Material

Pectoral spine, dentary, premaxillary.

Occurrences

Zones 1–2, 3, Mixed.

**Table:** 

*NOTURUS ELEUTHERUS* Jordan, 1877
*NOTURUS* SP. CF. *N*. *ELEUTHERUS* Jordan, 1877
MOUNTAIN MADTOM
Figure 5L

Material

Pectoral spine.

Occurrences

Zones 1–2, 4, Mixed.

**Table:** 

*NOTURUS STIGMOSUS* Taylor, 1969
*NOTURUS* SP. CF. *N*. *STIGMOSUS* Taylor, 1969
NORTHERN MADTOM
Figure 5M

Material

Pectoral spine.

Occurrences

Zone 3.

**Table:** 

*NOTURUS* SP. CF. *N*. *FLAVATER* Taylor, 1969
CHECKERED MADTOM
Figure 5J

Material

Pectoral spine.

Occurrences

Zones 1/2.

**Table:** 

*NOTURUS* SP. CF. *N*. *ELEGANS* Taylor, 1969
ELEGANT MADTOM
Figure 5K

Material

Pectoral spine.

Occurrences

Zones 1/2.

**Table:** 

GENUS *PYLODICTIS* Rafinesque, 1819
*PYLODICTIS OLIVARIS* (Rafinesque, 1818)
*PYLODICTIS* SP. CF. *P. OLIVARIS* (Rafinesque, 1818)
FLATHEAD CATFISH
Figure 5H

Material

Pectoral spine, articular, premaxilla, quadrate, ethmoid.

Occurrences

Zones 1–2, 3.

Remarks

There are 25 Ictaluridae considered native to the Tennessee River Basin (Etnier & Starnes, 1993; Boschung & Mayden, 2004), of which 6 were recently noted in TVA surveys of the mainstem. Overall, Ictaluridae are perhaps the most well-represented group in the Bell Cave assemblage. This is likely due to the preservation and identification bias of pectoral spines rather than indicating that this Pleistocene river system was actually dominated by Catfish. However, the comparatively large number of positively identified spine and cranial elements does convey their importance to the assemblage and to understanding this fish assemblage.

Ictaluridae elements were identified using comparative material and the descriptions and illustrations of individual elements from Taylor (1969), Lundberg (1982), Dickinson (1986), Thomas (2002) and Wright (2012). In particular, spines were identified by comparison of overall curvature, anterior and posterior serrae patterns, and insertion condyle shape relative to reference material. Of particular note regarding many of the described elements is the potential bias in identifications stemming from the effects of weathering and interspecific similarities among osteological patterns, such as similarities in spine serrae. These caveats likely suggest that our list is conservative compared to the true assemblage.

The most striking difference between modern mainstem diversity and Bell Cave diversity is in the Madtom group, which is entirely absent in recent standard mainstem fisheries surveys. The presence of the Madtom genus *Noturus* in Bell Cave collections provides evidence of a very different mainstem Pleistocene habitat compared with more recent habitats (currently modified into a chain of impoundments) as well as potential evidence for range expansions for several of these taxa. Madtom spines are often noted by their ornate serrae and curvature patterns coupled with their relatively small size. However, Stonecat Madtoms were distinguished primarily via a general lack of these ornamentations, including any anterior or posterior serrae, but with several posterior notches on the posterior distal end of the pectoral spine. Stonecat Madtom remains also included characteristic premaxillaries with elongated processes extending from functional tooth pads (Taylor, 1969; Lundberg, 1982; Dickinson, 1986). Conversely, *Rabida*-type spines were distinguished as having the typical elaborate or highly-ornamented posterior and anterior serrae patterns. Mountain Madtom was differentiated from Northern Madtom on the basis of less prominent anterior serrae (Thomas, 2002). Spines linked to the Northern Madtom are particularly unique as this taxon currently occurs in the Lake Erie and Ohio River drainage basins, well north of the Tennessee River Basin (Page & Burr, 2011). One caveat, however, is the historical lumping of Northern Madtom with the newly described Piebald Madtom (*N. gladiator*Thomas & Burr, 2004), which could have resulted in a bias of comparative collections and past descriptions. Checkered Madtoms were distinguished from other Madtoms (including the similar Brindled Madtom *N. miurus*) on the basis of their more prominent, recurved, and unique spacing of anterior serrae (Taylor, 1969). Interestingly, Checkered Madtom are considered an Ozark endemic today, yet given the Miocene divergence of this taxon (Hardman & Hardman, 2008) it seems that this contemporary pattern is best explained by the taxon’s extirpation from much of its former range. Dickinson’s (1986) identification of Checkered Madtom from Holocene deposits (not reported here) in Cheek Bend Cave support this hypothesis and indicate that the extirpation of this taxa occurred sometime after the Pleistocene.

Larger bodied taxa such as Channel Catfish, Flathead Catfish, and Bullhead Catfish were often more readily identifiable than Madtom due to their size and general lack of ornamentation compared to the smaller taxa. Channel Catfish were recognized from a variety of spine size classes using a general pattern of decreasing anterior serration size in more medial positions that tended to decrease in prominence in larger specimens (Lundberg, 1982). Bullhead Catfish were distinguished using serration groove features as well as noting similar-sized serrations along their posterior edge coupled with slight notches along the spine tip. Flathead Catfish were recognized primarily from spines exhibiting similar-sized and shaped serrae along both posterior and anterior spine edges (Lundberg, 1982).

Remains belonging to members of the Ictaluridae are diverse in Bell Cave, but have shown to be uncommon at other localities within our study area. Of the few reports, specimens identified as belonging to Ictaluridae have been recovered from site ACb-3 in Alabama (this study) and Cheek Bend Cave in Tennessee (Dickinson, 1986). Specimens identified as *Ictalurus* sp. have been identified at Baker Bluff (Guilday et al., 1978) and Beartown caves (Guilday, Hamilton & Parmalee, 1975) in Tennessee, and Channel Catfish has been identified from Bell Cave, Baker Bluff and Cheek Bend caves (Dickinson, 1986). Remains belonging to the Madtom group have been reported from sites ACb-3 and Baker Bluff, while remains of the Stonecat Madtom have been reported from only two localities, Bell Cave and Cheek Bend Cave. Bell Cave has also produced remains belonging to four additional Madtom species that have not been identified at any other locality within our study area, Mountain Madtom, Northern Madtom, Elegant Madtom, and Checkered Madtom. Bell Cave is also the only locality within our study area to produce late Pleistocene remains of Bullhead and Flathead Catfish (see Table 1).

**Table:** 

ORDER SCORPAENIFORMES Greenwood et al., 1966
FAMILY COTTIDAE Bonaparte, 1831
GENUS *COTTUS* Linnaeus, 1758
*COTTUS* SP.
SCULPIN
Figure 4N

Material

Premaxilla, dentary.

Occurrences

Zones 1–2, 3.

Remarks

Two Cottidae taxa are recognized natives of the Tennessee River Basin (Etnier & Starnes, 1993; Boschung & Mayden, 2004), the Mottled Sculpin (*Cottus bairdi*) and Banded Sculpin (*Cottus carolinae*). Both of these taxa are still recovered in mainstem fisheries surveys, albeit in relatively low numbers. The Sculpin fragments identified from Bell Cave lots were also some of the most uncommon of the taxa identified. Sculpin fragments were described based on characters outlined in Dickinson (1986) and Broughton (2000) which included recognition of lateral fossa, tooth arrangement, and generally narrow appearance along premaxilla and dentary pieces. Although the material is not definitive to the species level, we suggest these are the remains of the Banded Sculpin due to distribution and habitat preferences. Locating preopercles in the Bell Cave samples could potentially provide definitive identification to the species level (Broughton, 2000); unfortunately none were recovered.

Relative to the assemblage, the low abundances of Sculpin material might suggest something about the Pleistocene fish assemblage or it could reflect some sort of preservation bias. Today, Sculpin numbers are reduced in the mainstem as a function of habitat alterations occurring over the past century. Specifically, the reduction of riffle habitats in the modern Tennessee River mainstem has had implications for populations of these Sculpin taxa which key on gravel and rubble riffles (Boschung & Mayden, 2004) as an important part of their respective niches. In addition to the *Cottus* sp. specimens identified from Bell Cave, this taxon has also been identified from Appalachian Caverns in Tennessee (this study). Both Carolina Sculpin and Mottled Sculpin have been reported from Cheek Bend Cave in Tennessee (Dickinson, 1986; see Table 1).

**Table:** 

ORDER PERCIFORMES Bleeker, 1859
FAMILY CENTRARCHIDAE Bleeker, 1859
GENUS *AMBLOPLITES* Rafinesque, 1820
*AMBLOPLITES RUPESTRIS* (Rafinesque, 1817)
ROCK BASS
Figure 4M

Material

Dentary, premaxilla.

Occurrences

Zones 1–2, 3.

**Table:** 

GENUS *MICROPTERUS* Lacépède, 1802
*MICROPTERUS* SP.
BLACK BASS


Material

Dentary, premaxillary, maxillary, quadrate, angular, supracleithrum, infrapharyngeal, gill raker, operculum, vomer, hypohyal.

Occurrences

Zones 1–2, 3, 4, Mixed.

**Table:** 

*MICROPTERUS DOLOMIEU* Lacépède, 1802
*MICROPTERUS* SP. CF. *M. DOLOMIEU* Lacépède, 1802
SMALLMOUTH BASS
Figure 4K

Material

Dentary, premaxilla, pharyngeal, operculum.

Occurrences

Zones 1–2, 3, 4, Mixed.

**Table:** 

*MICROPTERUS* SP. CF. *M. PUNCTULATUS* (Rafinesque, 1819)
SPOTTED BASS
Figure 4L

Material

Dentary.

Occurrences

Zones 3.

Remarks

Watershed records as well as recent mainstem survey efforts include all four (three unequivocal) of these taxa as common native members of the modern Tennessee River fish assemblage. Interestingly, the most common Centrarchidae in recent mainstem collections are Bluegill (*Lepomis macrochirus*) and Largemouth Bass (*Micropterus salmoides*), neither of which were definitely identified from Bell Cave samples. The absence of Largemouth Bass and Bluegill is not particularly surprising given their habitat affinities for the more recent impoundment conditions of the Tennessee River mainstem that were nonexistent in the Pleistocene, coupled with decades of introduction efforts throughout the watershed. However, the absence of any Sunfish *Lepomis* taxa is surprising and should be further explored in future studies as several of these (e.g., see Table 1 list for Dust Bend and Cheek Bend Caves) are known from the basin during this time period and would be expected to have occurred in the Bell Cave assemblage. Diagnostic characters used to identify Centrarchidae taxa were primarily dentary and premaxilla shape differences. Specifically, Black Bass taxa tend to exhibit more uniform tooth size and arrangement patterns on all surfaces compared with either Rock Bass or Sunfish taxa. Diagnostic differences in the ‘shelf’ that runs along the dentary as well as premaxillary were also used to supplement tooth arrangement pattern identifications to delineate Black Bass species where possible (Dickinson, 1986).

Relative to other Pleistocene locales, specimens identified as unknown Centrarchidae have been reported from Saltville in Virginia (Dickinson, 1986). More specifically, Rock Bass appear extremely widespread across the Tennessee River Basin in the late Pleistocene at this taxa has been reported from Bell Cave (this study); Appalachian Caverns (this study), Baker Bluff (Guilday et al., 1978), Cheek Bend (Dickinson, 1986), and Guy Wilson (this study) caves in Tennessee, and Saltville in Virginia (Dickinson, 1986). Remains belonging to unidentified Black Bass have been reported from site ACb-3 in Alabama and Baker Bluff, while in addition to the remains from Bell Cave, specimens identified as Smallmouth Bass have been reported from Baker Bluff and Spotted Bass from site ACb-3 in Alabama (this study). A specimen identified as an unidentified Sunfish taxa has been reported from Dust Cave in Alabama while Green Sunfish have specifically been reported from Cheek Bend Cave. Remains belonging to this genus have yet to be identified from Bell Cave.

**Table:** 

FAMILY PERCIDAE Linnaeus, 1758
GENUS *SANDER* Oken, 1817
*SANDER* SP.
*SANDER VITREUS* OR *SANDER CANADENSIS*
WALLEYE OR SAUGER
Figures 5N–5O

Material

Teeth, dentary, premaxillary, angular, quadrate, maxilla, preoperculum, ceratohyal

Occurrences

Zones 1–2, 3, Mixed

**Table:** 

*SANDER VITREUS* (Mitchill, 1818)
*SANDER* SP. CF. *S*. *VITREUS* (Mitchill, 1818)
WALLEYE

Material

Teeth, dentary, articular, angular, premaxilla, epihyal, vomer, quadrate, palatine.

Occurrences

Zones 1–2, 3, Mixed.

**Table:** 

*SANDER* SP. CF. *S*. *CANADENSIS* (Griffith & Smith, 1834)
SAUGER

Material

Preoperculum, premaxillary

Occurrences

Zones 1–2.

Remarks

Both of these taxa are native to the Tennessee River drainage and have been surveyed in recent mainstem samples. Relative to modern mainstem TVA collections, the Sauger is a more numerically dominant member of the assemblage than the Walleye. If this trend has historical continuity it could mean that the majority of the unidentified remains should be assigned to Sauger (Griffith & Smith, 1834). The primary identification metric used to separate out the *Sander* genus from other remains were the characteristic and often diagnostic canine teeth, both loose and *in situ* within their robust and deep dentary sockets. Additionally, the singular row-arrangement of teeth compared with cluster arrangements found in other Perciformes (e.g., Centrarchidae) was used as a diagnostic feature. Differentiation between the two species was often a function of size inference (e.g., Walleye attain larger sizes than Sauger; Page & Burr, 2011) using measurements noted in comparative material. Of particular interest in these samples is the lack of material that could be assigned to other members of the family Percidae. For example, the Tennessee River drainage is currently home to 80+ other Percid species in the genera: *Etheostoma*, *Nothonotus*, *Percina*, and *Perca* (Etnier & Starnes, 1993; Boschung & Mayden, 2004; Page & Burr, 2011), several of which (12 taxa representing all major genera) are still collected in mainstem surveys (although *Etheostoma*/*Nothonotus* are likely underrepresented due to gear bias in recent TVA efforts). Specific to the Pleistocene samples, the lack of any Darter remains is likely a function of preservation, size, collection, recovery and processing biases inherent in the Bell Cave samples.

Three members of the Percidae family, Greenside Darter *E. blennioides*, Yellow Perch *Perca flacescens*, and Logperch *Percina caprode*s have been identified among the remains at Cheek Bend Cave in Tennessee (Dickinson, 1986). Remains of these three taxa have not been identified at Bell Cave nor have they been reported from any other locality within our study area. Remains identified as belonging to *Sander* sp. have been reported from Dust Cave in Alabama (Walker, 1998) and Baker Bluff Cave (Guilday et al., 1978) in Tennessee, while in addition to the Bell Cave specimens, Sauger has been reported from Baker Bluff and Cheek Bend caves, and Walleye from Cheek Bend Cave (see Table 1).

**Table:** 

FAMILY SCIAENIDAE Linnaeus, 1758
GENUS *APLODINOTUS* Rafinesque, 1819
*APLODINOTUS GRUNNIENS* Rafinesque, 1819
FRESHWATER DRUM
Figures 5P–5Q

Material

Pharyngeal, pharyngeal teeth, vertebrae, dentary, angular, spine, angular, parasphenoid, hypohyal, quadrate, unidentified skull fragments.

Occurrences

Zones 1–2, 3, 4, Mixed.

Remarks

The Freshwater Drum is the only Sciaenidae taxon found in freshwaters of North America and is extremely common in medium to large rivers throughout the Mississippi River drainage, including the Tennessee River. Recent TVA fisheries sampling efforts indicate that Freshwater Drum are one of the most abundant species (16/80) in the assemblage and likely higher in biomass than the majority of other taxa. This commentary on contemporary abundance appears to be consistent with the assemblages captured in the Bell Cave Pleistocene deposits as Drum samples were very well represented across all zones. Freshwater Drum remains, pharyngeal bones and teeth in particular, were identified from a variety of size classes and comprised the most prevalent materials in the Bell Cave samples. The pharyngeal bone was identified through robust thickness, triangular shape, and rounded tooth sockets of increasing circumference proximal to pharyngeal edges. Corresponding pharyngeal teeth are molariform in shape with rounded edges and a dome shaped crushing functional surface (i.e., bead or pearl shaped). Freshwater Drum pharyngeal bones and teeth are well-known in the Pleistocene fish literature; however, this represents the first associated museum records with collection information (but see Parmalee, 1992) for Alabama. In addition to the remains identified at Bell Cave, late Pleistocene remains of Freshwater Drum have been identified from site ACb-3 (this study) and Dust Cave (Walker, 1998) in Alabama, and Baker Bluff (Guilday et al., 1978) and Cheek Bend (Dickinson, 1986) caves in Tennessee (see Table 1).

## Discussion

The Tennessee River Basin fish assemblage preserved in the late Pleistocene deposits provide a detailed look into the paleoecology of one of the most species rich regions in North America. In total, 41 unequivocal taxa from 10 sites are reported from across the basin. Most significantly, excavations of Bell Cave deposits yielded the largest taxa list with a total of 11 families, 19 genera, and 24 identifiable fish species. Given that numerous uncommon fragment types could not be identified beyond family or genus level, this combined to indicate the presence of at least 28 unequivocal taxa in the assemblage.

The Bell Cave assemblage was captured in four snapshots denoted by ‘zones’ which ranged in age from approximately 10,000 to 30,000 years before present. None of the zones exhibited complete turnover in assemblages; however, during this 20,000 year range, Zone 3 (intermediate to Zones 1–2 and 4) was found to comprise the greatest diversity of unequivocal taxa. When grouped according to genera, diversity from Zones 1–2, 3, and 4 included 15, 15, and 8 distinct genera, respectively. Family diversity of Zones 1–2, 3, and 4 included 10, 11, and 8 distinct families, respectively. Of these families, 8 were common to each zone (Acipenseridae, Catostomidae, Centrarchidae, Cyprinidae, Esocidae, Ictaluridae, Lepisosteidae, and Sciaenidae), with the presence of 3 families (Percidae, Cottidae, and Anguillidae) driving the differences between zones. Although these zones are similar, differences between them may be attributable to habitat at the time of subaqueous deposition of Zone 4 and viscous mudflows resulting in Zones 1–2 and 3. This indicates full submersion of the cave habitat during the time period of Zone 4 with fish being stranded as mainstem water levels dropped compared with marginal riparian habitat flooding resulting in individuals washing and mixing with mudflows passing through fissures in the cave ceiling.

Not surprisingly, much of the contemporary basin wide fish diversity is not represented in the identified fragments or literature review—although the full complement of Tennessee River or basin wide fish diversity would not be expected to occur at a single site. This taxa diversity differential likely represents a taphonomic bias as a result of decomposition, transport, deposition, differential bone preservation, collection processing, or a combination of these factors. From what is preserved, the reconstructed Bell Cave assemblage resembles a typical medium-to-large river fish assemblage of the major contemporary Ohio River Basin tributaries (e.g., Wabash River; Jacquemin & Pyron, 2011). Compared with the modern Tennessee River Basin and specific Tennessee mainstem assemblage in the current locale (Alabama), a total of two and eleven taxa, respectively, are unique to the Bell Cave assemblage (Table 1; Appendices S1 and S2). This includes several taxa that have documented extirpations from the contemporary basin and at least one extinct species (e.g., Harelip Sucker). Overall, the assemblage found in Bell Cave is not vastly different from what is found in the Tennessee River Basin and mainstem today, as more than 20 taxa and all of the family/genera level groups are shared between the modern and late Pleistocene assemblages. The major differences between these assemblages seem consistent with natural post-Pleistocene dispersions and extirpations as well as modern effects of habitat changes (Boschung & Mayden, 2004).

These findings within the site and across the basin could help improve our understanding of why this region of the southeastern United States, especially the Eastern, Ozark, and Ouachita Highlands, exhibits such high diversity. Currently, regional diversity is explained using a combination of two hypotheses (see Ross & Matthews, 2014 for a review)—the dispersal hypothesis, which identifies the Eastern Highland region as the point of origin for lineages in the region that then dispersed along with glacial recessions into new drainages carved via outwash into their present distributions (Mayden, 1987; Strange & Burr, 1997) and the vicariance hypothesis, which suggests that lineages diverged in a single large Central Highland region that was then fragmented by the Mississippi River during the Pleistocene into the modern Ozark and Ouachita Highlands west of the Mississippi River and the Eastern Highlands east of the Mississippi River (Mayden, 1988). Numerous tests of these hypotheses have been undertaken in the past few decades using phylogeographic techniques and support for both dispersal and vicariance is well noted and considered specific to the lineage in question (Strange & Burr, 1997; Berendzen, Simons & Wood, 2003; Near & Keck, 2005).

In comparing faunas and drainage patterns for all of the study sites relative to modern habitat affinities and basin locales, these sites likely represent a combination of small to large stream systems (Fig. 1). For example, the presence of numerous Darter and Sculpin remains from several of the sites may indicate that the system was more likely a small-to-medium stream compared with a larger river (Page & Burr, 2011). Conversely, higher abundances of Walleye, Suckers, and Sturgeon would seem to point to a larger stream rather than a small to medium stream (Page & Burr, 2011). Based on these broad assemblage—habitat inferences, it seems likely that one of the most speciose sites, Cheek Bend Cave (25 unequivocal taxa), has preserved a small to medium stream compared with Bell Cave, situated along a much larger river. This inference is further supported when compared to modern site locales as Cheek Bend Cave sits along the much smaller Duck River, a tributary of the larger Tennessee River, on the banks of which is Bell Cave (see Fig. 1).

Despite the slight differences in taxa reported from all of the locations, the degree of taxa overlap throughout the Tennessee River Basin suggests a very similar fish composition was present during the late Pleistocene. As a practical caveat to these similarities across sites, fossil identifications are given multiple lines of evidence from multiple researchers. Interestingly, the two largest faunas, Bell Cave and Cheek Bend Cave, encompass almost every single taxon that could be identified at any of the other ten examined localities (with exception of Common Shiner and Bluntnose Minnow). In comparison of the faunas from all of the late Pleistocene sites with modern diversity, more than 75% of the represented taxa are listed as native and present within the Tennessee River Basin today (see Appendix S2).

These largely similar comparisons between Pleistocene and modern fauna are also paralleled in other taxa. Previous investigations of the Bell Cave (ACb-2) faunas have described the known birds (Parmalee, 1992), herpetofauna (Holman, Bell & Lamb, 1990; Jasinski, 2013), and mammals (Ebersole & Ebersole, 2011) from the site. Parmalee (1992) and Holman, Bell & Lamb (1990) described 39 bird taxa and 37 reptiles and amphibians, respectively, from the Bell Cave deposits. Of these 76 taxa, 34 of the birds and all 37 of the reptiles and amphibians have modern ranges that include the southern edge of the basin (e.g., northern Alabama) today. Of the birds, one extinct form was present, the Passenger Pigeon *Ectopistes migratorius*, an extinction resultant of human impact in historic times while four of the identified bird taxa from Bell Cave are not current residents in Alabama. Range changes in these bird taxa, including the Ruffed Grouse *Bonasa umbellus*, Greater Prairie Chicken *Tympanuchus cupido*, Common Raven *Corvus corax*, and Eurasian Magpie *Pica pica* are the result of Pleistocene dispersal (Parmalee, 1992). Ebersole & Ebersole (2011) published a detailed account of the Bell Cave mammals and recorded 55 taxa. Included among these mammals are 6 extinct megafauna—the Dire Wolf *Canis dirus*, Giant Beaver *Castoroides ohioensis*, Beautiful Armadillo *Dasypus bellu*s, Jefferson’s Ground Sloth *Megalonyx jeffersonii*, Long-nosed Peccary *Mylohyus nasutus*, and Vero Tapir *Tapirus veroensis*—and 16 extirpated taxa. These latter taxa are of two types, those which no longer have ranges in Alabama as a result of overhunting or habitat destruction (such as the Red Wolf, *Canis* sp. cf. *C. rufus*; North American Porcupine, *Erethizon dorsatum*; and Jaguar, *Panthera onca*), and those with boreal affinities that experienced northern dispersal patterns, such as the Red-backed Vole *Clethrionomys gapperi*, Least Weasel *Mustela nivalis*, Caribou *Rangifer tarandus*, and American Red Squirrel *Tamiasciurus hudsonicus*.

Taken together as a combined terrestrial and aquatic ecosystem, the presence of many of these mammals, birds, herpetofauna, and fishes suggest a more northern climate and habitat (e.g., boreal forest and streams) was present in this region during at least the late Pleistocene. This is further evidenced by the presence of extant stands of boreal trees in northern Alabama, such as hemlocks (Godfrey, 1988). The biogeographical implications of these occurrences suggest a dramatically different climate that recessed with several corresponding biota following the Pleistocene, leaving a few refugia to stand as evidence of this habitat. However, this conclusion is much more simplistic for vertebrate fauna generally more capable of dispersing over land than fishes that must follow waterways. Underlying the fish assemblage (see Appendices S1 and S2 for full species lists), the mechanism of faunal change is more nuanced. Several of the fishes noted in the study follow the dispersal hypothesis likely tracing climate north through the Mississippi River Basin as glaciers receded. Others seem to have been fragmented in populations from their original pre-Pleistocene Highland distributions more consistent with vicariance. Others still follow neither of these explanations and have experienced changes as a result of extinction.

Over 50 years ago, Hubbs & Miller (1948) very appropriately identified the lack of Pleistocene freshwater fish studies as a serious deficit contributing to our poor understanding of the biota associated with this geologic epoch relative to freshwater fish diversity and distribution. Given the paucity of literature pertaining to Pleistocene freshwater fishes, every taxon in this study (except those few mentioned without reference in Parmalee, 1992) represents a first recorded watershed occurrence or state record (particularly for Alabama) for this period. The implications of this study extend beyond state or river basin checklists, as it describes one of the single largest collections, both in lot number and identified unequivocal taxa, of described Pleistocene freshwater taxa in the southeastern United States.

However, the presence of 41 unequivocal taxa reported here from late Pleistocene localities within the Tennessee River Basin compared with modern diversity records suggests the incidence of significant biases as a result of any or all of the following: preservational, taphonomic, collection, and identification biases. Nevertheless, the assemblages discussed herein (particularly, Bell Cave and Cheek Bend Cave) represent the most intact Pleistocene freshwater fish deposits in North America. Future investigation can hopefully reduce these biases and add to the work currently presented here. In particular, as molecular techniques continue to improve, it may be possible to amplify ultrashort DNA fragments that could remain in the preserved bones (Dabney et al., 2013). Molecular work would not only help to identify fragmentary remains, but could also better establish genetic linkages between contemporary and Pleistocene populations and further explore the role that dispersal and vicariance played in establishing the modern Highlands region. Overall, these future efforts could elicit additional samples of underrepresented taxa as well as potentially recover unknown species providing ecological, evolutionary, and biogeographical implications.

## Supplemental Information

10.7717/peerj.1648/supp-1 Supplemental Information 1 Appendix Material Raw data.

10.7717/peerj.1648/supp-2 Supplemental Information 2 Table 1

## Additional Information and Declarations

### Data Availability

The following information was supplied regarding data availability: All raw data is provided as Supplemental Information 1.
